# Dovitinib synergizes with oxaliplatin in suppressing cell proliferation and inducing apoptosis in colorectal cancer cells regardless of RAS-RAF mutation status

**DOI:** 10.1186/1476-4598-13-21

**Published:** 2014-02-04

**Authors:** Shikha Gaur, Linling Chen, Vincent Ann, Wei-Chen Lin, Yafan Wang, Vincent HS Chang, Nan Yong Hsu, Her-Shuyong Shia, Yun Yen

**Affiliations:** 1 Department of Molecular Pharmacology, Beckman Research Institute of the City of Hope, Duarte, CA 91010, USA; 2 Translational Research laboratory, Beckman Research Institute of the City of Hope, Duarte, CA 91010, USA; 3 Institute of Translational Medicine, Taipei Medical University, 250 Wu-Hsing Street, Taipei 110, Taiwan; 4 Eugene and Ruth Roberts Summer Academy of City of Hope, Duarte, CA 91010, USA; 5 City of Hope Comprehensive Cancer Center, Beckman Center, Room 4117, 1500 E. Duarte Road, Duarte, CA 91010, USA

**Keywords:** Colorectal cancer, Cell proliferation, Apoptosis, Signal transduction, Immunohistochemistry, Ki-67, Caspases, Chemotherapy

## Abstract

**Background:**

Cancer is the result of a multistep process of genomic alterations, including mutations in key regulatory proteins that result in loss of balanced gene expression and subsequent malignant transformation. Throughout the various stages of colorectal carcinoma (CRC), complex genetic alterations occur, of which over-expression of growth factors, such as vascular endothelial growth factor, fibroblast growth factor and platelet-derive growth factor and their corresponding receptor tyrosine kinases, have been shown to correlate with invasiveness, tumor angiogenesis, metastasis, recurrence, and poor prognosis of colorectal cancer. To evaluate the therapeutic effect, we combined Dovitinib, an orally bioavailable, potent inhibitor of class III-V receptor tyrosine kinases with chemotherapeutic drug, oxaliplatin in preclinical models of colon cancer.

**Methods:**

Human colon cancer cells with different RAS-RAF mutation status (HCT-116, HT-29, SW-480, CaCO2 and LS174T) were treated with a combination of Dovitinib and Oxaliplatin at low dosage followed by assays to investigate the effect of the combination on cell proliferation, cell migration, cell apoptosis and signaling pathways involved in molecular mechanism of drug(s). The antitumor effects of either of the drugs were compared to the combination using human colon carcinoma cell line HT-29 xenograft model. Treated vs untreated tumor sections were also compared for proliferation and angiogenesis markers by immunohistochemistry.

**Results:**

The combination of dovitinib and oxaliplatin showed higher *in vitro* cytotoxicity in colon cell lines irrespective of their RAS-RAF status as compared to either of the drugs alone. Simultaneous inhibition of MAP kinase and AKT pathways and induction of apoptosis via activation of caspases 9/caspases 3 contributed to the synergistic effect of this combination therapy. In the xenograft model, the combination showed a significantly higher antitumor activity. Immunohistochemistry of post treatment tumors showed a significant decrease in proliferation and angiogenesis as compared to either of the treatments alone.

**Conclusions:**

This study demonstrates the synergistic antitumor activity of combination of dovitinib and oxaliplatin against colon cancer with different RAS-RAF status. The combination also showed its antitumor efficacy in a multidrug resistant phenotype xenograft model. This provides a basis for further investigation for its potential in clinical setting for colorectal cancer.

## Background

Colorectal cancer (CRC) is the third most common cancer in men (663,000 cases, 10.0% of the total) and the second in women (571,000 cases, 9.4% of the total) worldwide (Globocan, an international agency for research on cancer). American cancer Society estimates that in 2013, approximately 142,820 new cases of colorectal cancer will be diagnosed with 50,830 (26,300 men and 24,530 women) in United States alone. Overall, the lifetime risk of developing colorectal cancer is about 1 in 20 (5.1%) [1,2]. A number of different drugs have shown significant antitumor activity in CRC, including the systemic drugs 5-fluorouracil (5-FU), irinotecan, oxaliplatin, bevacizumab, cetuximab and panitumumab, and the oral drug capecitabine. Different regimens of these drugs, such as the FOLFOX (leucovorin, 5-FU and oxaliplatin), FOLFIRI (leucovorin, 5-FU and irinotecan) and XELOX (oxaliplatin and capecitabine), with or without a monoclonal antibody agent have shown improved outcomes in CRC [3-10]. The efficacy of chemotherapy has reached a plateau and a 5-year survival rate of patients with advanced CRC still remains < 8% [11] with the underlying molecular basis still not clearly defined.

With the advancement of genomic technology and availability of various genetic animal models, it has been proposed that the progression of CRC is from cumulative changes in key genes controlling cell proliferation, apoptosis and invasion [12-17]. Abnormally high activation of multiple signaling pathways such as RAS-RAF, and WNT/APC/β-Catenin has been demonstrated to be required for initiation and progression of colorectal carcinoma [18-20]. Some of these pathways are regulated by key enzymes known as tyrosine kinases which phosphorylate tyrosine residues in protein that are associated with either transmembrane receptor-linked proteins or non-receptor cytoplasmic proteins [21]. Activated forms of these enzymes are known to increase tumor cell proliferation and growth, induce antiapoptotic effects and promote angiogenesis and metastasis [22]. In addition to activation by growth factors, kinase activation by somatic mutations is also a common mechanism for tumorigenesis [23]. Mutations in *kRAS* (31%) and *BRAF* (9.6%) are both thought to occur early in colorectal carcinogenesis and are associated with significantly poor survival [24,25]. Although majority studies show that these two mutations are rarely observed together, a recent study in Chinese patients with CRC showed approximately 25% of the population harboring both kRAS and bRAF mutations [26]. The presence of multiple mutations has always posed potential limitations to the inhibitors. Since receptor tyrosine kinase activation initiates these effects, they are the key targets for inhibitors [22,27]. The majority of currently available tyrosine kinase inhibitors has provided a new approach for cancer therapy and has the potential for avoiding some of the drawbacks of cytotoxic chemotherapy [22]. Targeted agents have also offered an opportunity to reverse chemotherapy resistance and enhance response in patients with localized or advanced cancer [28]. Along with holding a great promise, these inhibitors have also posed drawbacks, being beneficial to only certain subpopulations of patients and limiting resistance in patients who initially responded [29-31].

Dovitinib, or TKI258 (4-amino-5-fluoro-3-[5-(4-methylpiperazin-1-yl)-1H-benzimidazol-2-yl]quinolin-2 (1H)-one; formerly known as CHIR-258), is a small molecule adenosine 5′-triphosphate–competitive inhibitor of class III, IV, and V receptor tyrosine kinases (RTKs), which include fibroblast growth factor receptor (FGFR), vascular endothelial growth factor (VEGFR), Tyrosine-protein kinase kit (c-KIT), and FMS-like tyrosine kinase 3 (FLT3) [32-35]. According to previous studies, dovitinib exhibits potent tumor growth inhibition *in vitro* and in a broad range of preclinical animal models [32,36-38]. For example, dovitinib induced apoptosis in Fibroblast growth factor receptor (FGFR) expressing mammary cells via inhibition of Phosphoinositide-3-kinase (PI3K)/Akt signaling pathway [39]. In addition, dovitinib specifically inhibited proliferation and survival of primary cells and cell lines with FGFR1 fusion genes associated with the 8p11 myeloproliferative syndrome [40].

There remains a need for not only novel regimens but also refinement of existing regimens to improve and extend survival and decrease treatment related toxicities. In the present study, we hypothesized that Dovitinib may attempt to boost therapeutic kill by employing combination regimen with oxaliplatin. Our results reveal that co- treatment of Dovitinib and Oxaliplatin in colon cancer cell lines induced superior cell killing in comparison to either of these drugs alone in all colon cancer cell lines regardless of their mutation status. The significantly enhanced antitumor activity that results from the combination of Oxaliplatin and Dovitinib offers promise as a novel treatment for patients with colon cancer. This combination will achieve a greater anticancer effect at a lower efficacious dose with a less chance of a cell developing resistance along with reduced injury to normal cells.

## Results

### Combination of Dovitinib and Oxaliplatin inhibits cell viability and migration in colorectal carcinoma cell lines

**Figure 1:**
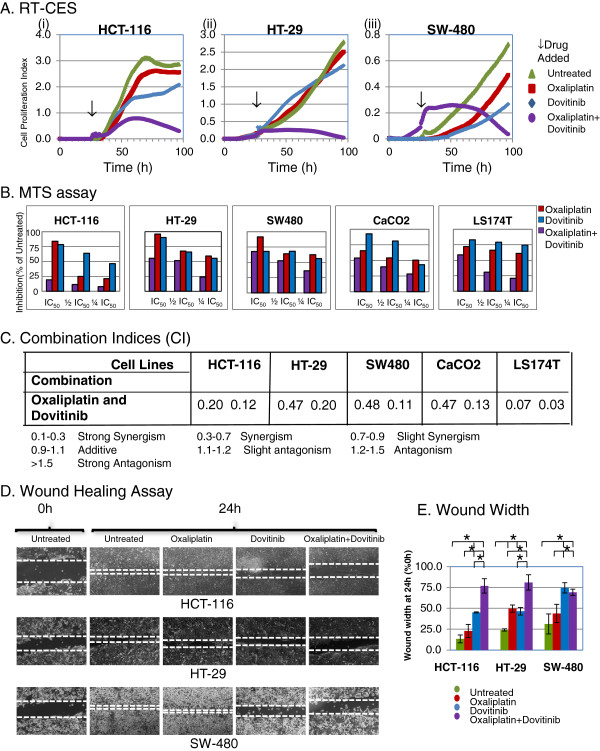
**Effect of dovitinib and/or oxaliplatin on growth and migration of tumor cells *in vitro*.** Combination of Dovitinib and Oxaliplatin produce synergistic effect in colon cancer cell lines: **(A)**, Representative Panel of combination effect measured by RT-CES **(B)**, a representative experiment plotted as histogram showing an effect of oxaliplatin and/or dovitinib in cell viability using MTS assay colon cancer cell lines. **(C)** Combination index calculated using Biosoft calcusyn. The values represent Mean ± SEM for at least 3 independent populations measured in triplicates. **(D)** Images of migration of cells 24 h after injury in the presence or absence of dovitinib and/or oxaliplatin in colon cancer cell lines. **(E)**, Percentage of wound closure at 24 h. The graph indicates Mean ± SEM of wound closure at 24 h (*n* = 3). *P < 0.01. Combination of dovitinib and oxaliplatin inhibits cell proliferation and migration in colon cancer cells.

We performed MTS assay to find out the combined effect of dovitinib and oxaliplatin in colon cancer cell lines. Several human colon cancer cell lines (HCT-116, HT-29, SW-480, Caco2 and LS174T) were tested for antiproliferative effects of individual drugs after 72 h of incubation. Table 1 summarizes the half maximal inhibitory concentration (IC_50_) values (mean ± standard deviation) and mutation status for different cell lines. All cell lines showed sensitivity to dovitinib in low micro molar range (3-5 μM) and sensitivity to oxaliplatin varied from 1.6 ± 0.17 μM to 8.0 ± 2.0 μM. HCT-116 cell line showed highest sensitivity to both the drugs among all five colorectal cancer cell lines tested. HT-29 cells showed the least sensitivity to both the drugs probably due to the presence of p-glycoprotein or multidrug resistance protein efflux pump [41]. These results are in agreement with previously published IC_50_ values of dovitinib in breast, bladder and pancreatic cancer cell lines and oxaliplatin in colon, breast and pancreatic cancer cell lines. Thus, the majority of colon cancer cell lines revealed sensitivities similar to or slightly better than most other cancer cell lines. Figure 1A shows the effect of dovitinib and/or oxaliplatin over real time in HCT-116, HT-29 and SW-480 cells as recorded by RT-CES (Real time cell electronic sensor). Both dovitinib and oxaliplatin inhibited the cell growth in HCT-116 and SW-480 cells and insignificant change in HT-29 with either of the drugs alone. However, the combined effect of dovitinib and oxaliplatin was more pronounced as compared to either of the drugs alone in all cell lines. In HT-29 cells addition of dovitinib may have sensitized the cells to oxaliplatin. Combination of two drugs added simultaneously showed better cytotoxicity as compared to sequential (oxaliplatin-dovitinib or dovitinib-oxaliplatin) addition (data not shown). The RT-CES data was confirmed by 3-(4,5-dimethylthiazol-2-yl)-5-(3-carboxymethoxyphenyl)-2-(4-sulfophenyl)-2H-tetrazolium (MTS) assay. The combination of dovitinib and/or oxaliplatin in all five colon cancer cell lines is shown as bar diagram (Figure 1B) and the calculated combination indices (CI) for the combined effect is summarized in Figure 1C. Our results show a strong synergistic effect of the combination despite different mutation status among these cell lines. LS-174 T and HCT-116 showed the strongest synergistic effect of the dovitinib and oxaliplatin combination in comparison to HT-29, SW-480 and Caco2 cells.

**Table 1:** Mutation status and IC50(μM) for Dovitinib and Oxaliplatin in Colon cancer cell lines

**Cell Lines**	**HCT-116**	**HT-29**	**SW-480**	**CaCO2**	**LS174T**
***Status***					
**Drugs**					
**KRAS**	**M(Gly13Asp)**	**WT**	**M(Gly12Val)**	**WT**	**M(Gly12Asp)**
**BRAF**	**WT**	**M(Val600Glu)**	**WT**	**M(Val600Glu)**	**WT**
**PIK3CA/pTEN**	**M**	**WT**	**WT**	**WT**	**M**
**p53**	**WT**	**M (273Arg-His)**	**M (273Arg-His**	**M**	**WT**
			**309Pro-ser)**		
**LOH/MSI**	**MSI**	**MSS/LOH**	**MSS/LOH**	**MSS**	**MSI**
**APC**	**WT**	**M**	**M**	**M**	**WT**
**Dovitinib**	**3.050.58**	**5.21.93**	**4.330.47**	**3.230.64**	**4.330.47**
**Oxaliplatin**	**1.670.17**	**8.02.0**	**6.02.08**	**2.70.15**	**2.871.27**

*Abbreviations*: *M* Mutant, *WT* Wild type, *LOH* Loss of heterozygosity, *MSI* Microsatellite instability, *MSS* Microsatellite stable.

Using the wound healing assay, we next examined the cancer cell migration in response to mechanical wound. Figure 1D shows a representative picture of HCT-116, HT-29 and SW-480 cells in monolayer culture after subject to mechanical scratch wound injury in the absence or presence of dovitinib (1 μM) and/or oxaliplatin (5 μM) before and after 24 h drug treatment. The width of cell-free wound zone at the end of 24 h post-injury period was measured and expressed as% of wound width at 0 h (t_0_). At the end of 24 h (t_24_), 86.7% ± 4.7, 76% ± 1.4 and 68.8% ± 12.0 wound was resealed in untreated HCT-116, HT-29 and SW-480 respectively. Oxaliplatin showed a significant difference in migration of cells in HT-29 (50.3% ± 4.3, p < 0.001) when compared to untreated cells. Dovitinib treatment alone inhibited the wound width by approximately 55% (p < 0.001) in HCT-116 and HT-29 as compared to 75% (p < 0.01) in SW-480 cells. The combination of dovitinib and oxaliplatin inhibited cell motility by approximately 75% (p < 0.001) in HCT-116 and HT-29 cell lines while no additional inhibition was observed in combination group in SW-480 cells. Treatment of cultures with two drugs resulted in a significant inhibition in cell migration compared to untreated cultures (Figure 1E).

### Combination of Dovitinib and Oxaliplatin inhibits its cellular targets

**Figure 2:**
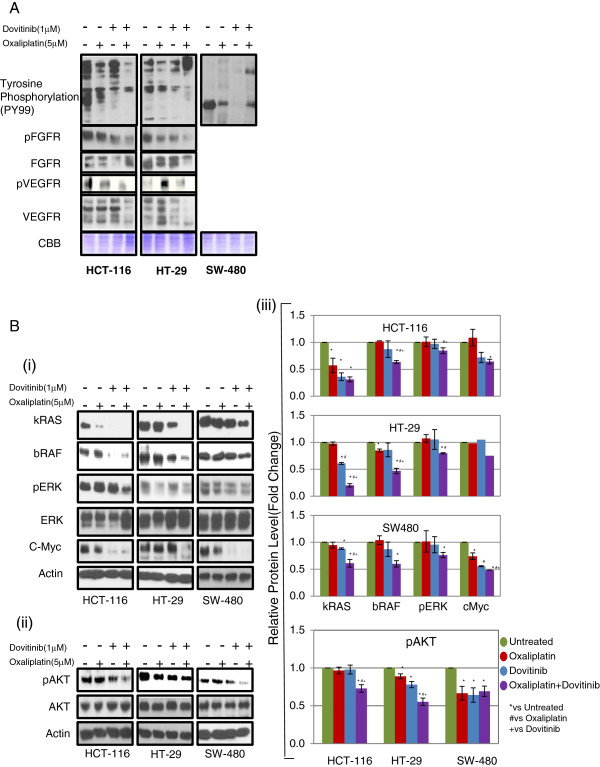
**Effect of dovitinib and/or oxaliplatin on MAP Kinase and PI3Kinase pathway.** Western blot analysis of phosphorylation levels after treatment with combination of dovitinib and oxaliplatin in colon cancer cell lines. The cells were starved for 24 h followed by treatment with 10 ng/ml FGF for 30 min prior to the addition of either of the drugs for 24 h. **(A)** Representative protein bands for tyrosine phosphorylation (py99), p-FGFR and pVEGFR. pFGFR and pVEGFR expression levels were decreased at 24 h after treatment. Coommassie blue (CBB) was used to show equal protein loading in each lane. **(B)** Representative protein bands for kRAS, bRAF, pERK, ERK and cMyc **(i)** and pAKT and total AKT **(ii)** signaling pathways. β-actin was used to show equal protein loading in each lane. **(iii)** Bar diagram (Mean ± SEM) showing the fold change in the intensity of proteins with respect to b-actin and normalized to untreated group.  = 3; *#+  < 0.05, vs Untreated, # vs Oxaliplatin and + vs Dovitinib respectively. Combination of dovitinib and oxaliplatin show more pronounced inhibition in expression of proteins involved in MAP kinase and PI3Kinase pathway.

To elucidate the underlying mechanism by which combination of oxaliplatin and dovitinib induces inhibition of proliferation, we examined the alterations in signal transduction pathway induced by oxaliplatin and/or dovitinib in colorectal cancer cell lines. As stated, dovitinib has been shown to be a potent inhibitor of receptor tyrosine kinases (RTK’s) including FGFR, VEGFR2 and PDGFR. We determined the overall tyrosine phosphorylation pattern in cell lysates prepared from oxaliplatin and/or dovitinib treated cells. Oxaliplatin showed insignificant changes in the phosphorylation pattern while treatment with dovitinib inhibited the phosphorylation of several proteins in all three cell lines. Next, we checked the expression levels of receptors in the presence of oxaliplatin and/or dovitinib in all three CRC cell lines by western blot analyses. Only HCT-116 and HT-29 were found to have expression of both receptors. These two cell lines showed insignificant changes in the phosphorylation of FGFR and VEGFR after treatment with either oxaliplatin or dovitinib while the combination of the drugs showed a significant down-regulation of phosphorylation of both the receptors (Figure 2A). Since SW480 cells showed absence or very low expression most of these receptors, we checked the effect of the combination on downstream signalling pathway proteins.

RAS proteins are small GTPases known for their involvement in oncogenesis. Around 25% of human tumors present mutations in a member of this family [42]. Many studies have cleared that activating mutations in members of the *RAS* family of genes are among the most common genetic lesions in human tumors and these mutations lock RAS proteins into a constitutively activated state in which they signal to downstream effectors even in the absence of extracellular stimuli. Involvement of RAS signaling in cancer is accentuated by the incidence not only of *RAS* mutations but also the deregulation of many of its regulators or effectors pathways [43,44]. The first RAS effector pathway to be identified was the RAF-MEK-ERK pathway [45]. Next, if RAS or RAF mutations play any role in the response to the treatment with oxaliplatin and dovitinib, we determined the expression levels of these proteins in the presence/absence of these drugs. Irrespective of RAS-RAF mutations, dovitinib inhibited the expression of both RAS and RAF in all three cell lines tested. The combination of the two drugs showed an even more pronounced inhibition of both RAS and RAF proteins. In addition, dovitinib inhibited phosphorylation of ERK, a downstream signaling molecule of RTKs. These data indicate that dovitinib inhibits the activity of its target receptors in tumor cells and results in down modulation of the signaling pathway (Figure 2B (i) and (iii)).

The second best-characterized RAS effector family is phosphoinositide 3-kinases (PI3Ks), which play important roles as mediators of RAS-mediated cell survival and proliferation. When active, PI3K converts phosphatidylinositol (4, 5)-bisphosphate (PIP_2_) into phosphatidylinositol (3, 4, 5)-trisphosphate (PIP_3_). PIP_3_, in turn, binds the pleckstrin homology (PH) domain of Akt/PKB, stimulating its kinase activity, resulting in the phosphorylation of a host of other proteins that affect cell growth, cell cycle entry, and cell survival [42,46]. Next, we determined the phosphorylation of AKT in response to treatment with oxaliplatin and/or dovitinib. Oxaliplatin or dovitinib produced insignificant inhibition in AKT phosphorylation in HCT-116 cells, however the phosphorylation decreased significantly in combination group as compared to untreated or either of the treated cells. Dovitinib inhibited the phosphorylation in HT-29 cells which decreased further after the combined treatment. SW-480 cells showed a decrease of phosphorylation in all three treatment groups (Figure 2B (ii) and (iii)).

### Combination of Dovitinib and Oxaliplatin induces DNA damage

**Figure 3:**
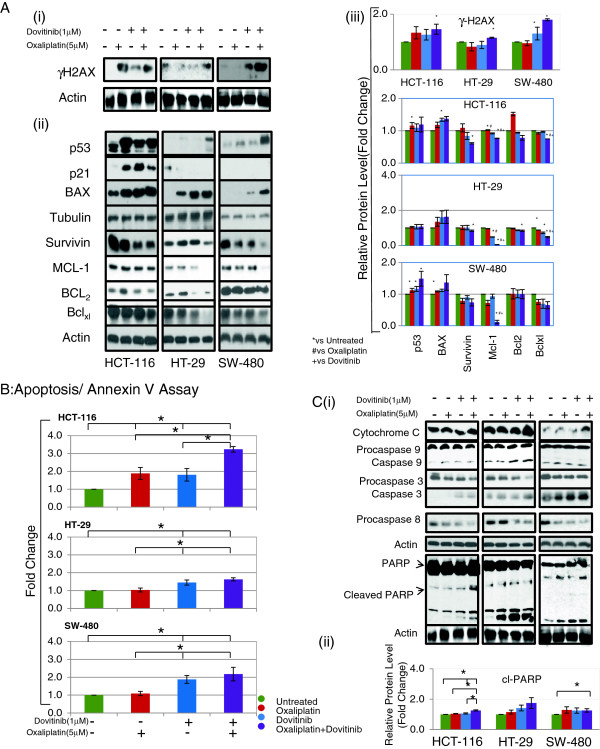
**Effect of dovitinib and/or oxaliplatin onset of tumor cell apoptosis and caspases activation.** Western blot analysis of DNA damage and proteins involved in apoptotic pathway after treatment with combination of dovitinib and oxaliplatin in colon cancer cell lines **(A)** Expression of γH2AX **(i)** and p53, BAX, Blc-2, Bclxl, Mcl-1 and survivin at 24 h after treatment **(ii)**. γ-tubulin or β-actin was used to show equal protein loading of each lane. **(iii)** Bar diagram (Mean ± SEM) showing the fold change in the intensity of proteins with respect to β-actin or γ-tubulin and normalized to untreated group.  = 3; *#+  < 0.05, vs Untreated, # vs Oxaliplatin and + vs Dovitinib respectively. **(B)** Cell cycle analysis: approximately one million cells before and after treatment with dovitinib and/or oxaliplatin for 48 h. Cells were harvested, stained with annexin V–FITC and/or propidium iodide and subjected to flow cytometric analysis. The percentage of annexin V–FITC positive cells were determined by calculating the ratio to the total number of cells. Each bar represents the Mean ± SEM of at least three independent experiments, *p < 0.05. **(C)** Induction of caspases cascade and PARP after treatment with dovitinib and/ or oxaliplatin **(i)**. Bar diagram (Mean ± SEM) showing the fold change in the intensity of cleaved PARP with respect to β-actin and normalized to untreated group.  = 3; *  < 0.05 **(ii)**. Combination of dovitinib and oxaliplatin show up-regulation of proapoptotic and down-regulation of antiapoptotic proteins via caspases dependent apoptotic pathway.

A very early step in the cellular response to chemotherapeutic drugs is DNA double strand breaks (DSBs) followed by the phosphorylation of a histone H2A variant, H2AX, at the site of DNA damage. We determined the extent of DNA damage by adding oxaliplatin and dovitinib. The phosphorylation of H2AX increased in HCT-116 cells after treatment with individual drugs but the combination of two drugs did not show any additional increase. Oxaliplatin or dovitinib alone produced a negligible change in the phosphorylation status in HT-29 and SW-480 cells, however, these cells showed a clear increase in phosphorylation with the combination treatment (Figure 3A (i) and (iii)).

### Combination of Dovitinib and Oxaliplatin up-regulates expression of pro-apoptotic proteins with concomitant decrease in levels of anti-apoptotic proteins

The pathway leading to induction of apoptosis also involves p53, where p53 may act in association with other proteins such as Bax (proapoptotic) and Bcl2 family (antiapoptotic). We next assayed the expression levels of p53, Bax, Mcl-1, Survivin, Bcl_xl_ and Bcl2 in the presence of oxaliplatin and/or dovitinib. Figure 3A (ii) shows that treatment of HCT-116, HT-29 and SW-480 cells with the combination of the two drugs for 24 h up-regulated the expression of p53 and Bax. However, the expression of Bcl2, Mcl-1, Survivin and Bcl_xl_ decreased significantly in all three colon cancer cell lines when treated with the combination of oxaliplatin and dovitinib. The effect of combination was more pronounced in both proapoptotic and antiapoptotic proteins as compared to either of the drugs alone. The quantitation intensity of each protein band in Figure 3A (i) and (ii) with respect to β-actin or γ-tubulin is shown in Figure 3A (iii).

### Combination of Dovitinib and Oxaliplatin induce apoptosis in colorectal carcinoma cell lines

Through quantitative apoptotic cell death assay, we assessed whether the observed synergistic cell growth inhibition by oxaliplatin-dovitinib combination is accompanied by greater apoptotic cell death. Figure 3B shows combined population of proapoptotic (annexin V positive) and apoptotic cell (PI positive) population. Oxaliplatin and dovitinib individually increased the proapoptotic cell population by 1.8 fold in HCT-116 cells but the combination increased this number to approximately 3fold. HT-29 and SW-480 showed no cell death in the presence of oxaliplatin alone and only marginal increase in annexin positive cells with dovitinib. However, the combination of two drugs increased this number to 1.6 and 2.2 fold in HT-29 and SW-480 cell respectively. Combination of oxaliplatin and dovitinib increased the PI positive cell population by 1.5 to 1.8 fold (p = 0.05) as compared to untreated cells in all three colon cancer cell lines tested.

To study the mechanistic aspects of apoptosis induction by the combination of oxaliplatin and dovitinib, we next assessed the activation of caspases by Western blot analysis. Although treatment with oxaliplatin or dovitinib alone increased the levels of cleaved caspase-9 and -3 in all three cell lines tested, the increase was more amplified with the combination of the two drugs indicating the involvement of these caspases in apoptosis induction (Figure 3Ci). An important factor in inducing apoptosis is the enzyme Poly (AD ribose) polymerase (PARP) that detects DNA strand breaks and functions in base excision repair. Once PARP is cleaved, it no longer supports the enzymatic DNA repair function. It is a known marker of apoptosis and a downstream target of activated caspase-3 [47]. The expression of cleaved PARP was greatly enhanced after the treatment of HCT-116, HT-29 and SW-480 cells with combination of oxaliplatin and dovitinib, which might have contributed to the commitment to apoptosis (Figure 3C (i)). The quantitation intensity of PARP with respect to β-actin is shown in Figure 3C (ii).

### Combination of Dovitinib and Oxaliplatin inhibits tumor growth in Human Colorectal Cancer HT-29 Xenograft

**Figure 4:**
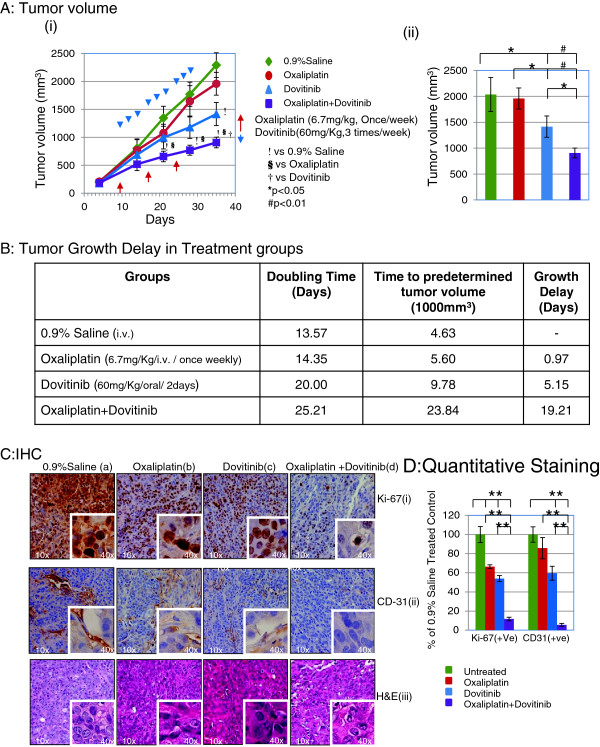
**The antitumor effect of dovitinib and/or oxaliplatin on the xenograft of colon cancer.** Treatment with dovitinib and oxaliplatin significantly decrease the growth of HT29 colon cancer xenografts in athymic mice. In these experiments, mice were treated with 60 mg/kg dovitinib (oral/2 days) and/ or 6.7 mg/kg oxaliplatin (i.v., once/week). Matched control mice bearing xenografts received an equal volume of saline on the same treatment schedule. Significance was determined using a two-way ANOVA. **(Ai)** Tumor volume changes after administration of drugs. **(Aii)** Bar diagram (Mean ± SEM) showing the tumor volume on day 28 after the treatment,  = 10; *  < 0.05 and #p < 0.01. **(B)** Tumor growth delay in various treatment groups calculated from a Log-linear growth plot. **(C)** Representative panel of immunohistochemical analysis of **(i)** Ki-67 and **(ii)** CD-31 (cluster of differentiation molecule also known as PECAM-1) in tumors extracted from mice with/without treatment with dovitinib and/or oxaliplatin. Magnification (10×) with inset at (40×). **(iii)** H & E staining of corresponding sections from untreated and treated tumors. **(D)** Bar diagram (Mean ± SEM) showing the quantitation of average number of Ki-67 positive cells and CD-31 positive vessels as percentage of saline treated control,  = 10; *  < 0.05. Combination of dovitinib and oxaliplatin inhibits the tumor growth by inhibition of cell proliferation and angiogenesis.

Based on our results showing strong efficacy of the combination of oxaliplatin and dovitinib in colorectal cancer cells in culture, next we examined the *in vivo* efficacy of the combination against the colorectal cancer HT-29 xenograft in athymic nude mice. Figure 1A (i) shows tumor growth curve after treatment with oxaliplatin and/or Dovitinib. Treatment with 3 weekly doses of oxaliplatin (day 11, 18, and 25) at 6.7 mg/Kg was inactive as monotherapy in all animals. This is in agreement with a study showing inactivity of oxaliplatin as monotherapy in HT-29 animal model at MTD of 10 mg/Kg [48]. Similarly an alternate day dose of 60 mg/Kg Dovitinib showed a decrease of tumor growth as early as day 4 after treatment in all animals. The combination of two drugs showed a significant decrease (p < 0.05) in tumor growth stating from an early stage as compared to vehicle or oxaliplatin treatment while at late stage compared to dovitinib alone. Similar results were also observed by Lee *et al.* in KM12L4a and HCT-116 colon cancer tumor models at dose level of 30-100 mg/Kg [32]. The average final tumor volume was reduced from 2,036 ± 327.5 mm^3^ in control group to 1,957 ± 204.0 and 1,415 ± 205.9 mm^3^ in oxaliplatin and dovitinib treatment groups respectively, accounting for only 4% decrease in oxaliplatin group and 31% (*P* < 0.001; Figure 4A (ii)) decrease in dovitinib treatment group at the end of the experimental period. However, the combination treatment group showed a tumor volume of 906 ± 94.8 mm^3^, an approximate 55% decrease in tumor volume from vehicle or oxaliplatin group and 36% decrease from dovitinib treatment group. Tumor growth delay and doubling time (DT) of tumor volume of each group was calculated from a Log-linear growth plot (Figure 4B). The tumor growth delay was calculated as the time difference between each treatment group and the control group when the average tumor size reached 1000 mm^3^. Combination treatment with oxaliplatin and/or dovitinib did not show any gross signs of toxicity and/or possible adverse side effects as measured by two profiles, that is, body weight and diet consumption. Also, the necropsy report showed no abnormality in these mice at the end of the experiment. Reports have shown that Dovitinib as high as 120 mg/Kg for continuous 25 days exerted no toxicity in animals [32].

In an attempt to understand some of the details of the mechanism of action of combination, the tumors were removed from mice and processed for immunohistochemical expression of Ki-67 and CD-31. Ki-67 antigen is the prototypic cell cycle related nuclear protein, expressed by proliferating cells in all phases of the active cell cycle and absent in resting cells. It is routinely used as a marker for proliferating cells. Representative photomicrographs of Ki-67 antigen–stained sections from untreated, oxaliplatin and/or dovitinib tumors are shown in Figures 4C (i) a-d. Staining for Ki-67 decreased immensely with the treatment of oxaliplatin combined with dovitinib compared to untreated tumors as well as either of oxaliplatin or dovitinib administered groups.

Angiogenesis is crucial for tumor development and CD31 is widely used as a marker to highlight the density of intra-tumoral vessels and the degree of neoangiogenesis. Its immunoexpression was mainly localized in the junction between cells and is clearly positive in tumors from the vehicle control group with slight inhibition in the oxaliplatin and dovitinib treated tumors and almost negligible staining in combination treatment group (Figure 4C (ii) a-d). Figures 4C (iii) a-d show the H & E staining in tumors from all the groups. The quantitative data for immunostaining is shown in Figure 4D.

These results suggest an *in vivo* antitumor efficacy of the combination against colorectal cancer without any apparent signs of toxicity. Also, antitumor effect of the combination of oxaliplatin and dovitinib is due to inhibition of both proliferation and angiogenesis.

## Discussion

In this study, we evaluated the growth inhibitory effects of dovitinib and oxaliplatin combination in cell culture and xenograft models of colon cancer, and our goal for this investigation was to elucidate potential molecular mechanisms of action for the compounds contributing to the antiproliferative and anticancer capacity of human colon cancer cells. We found that both oxaliplatin and dovitinib (in the low micromolar range) effectively diminished the growth of colon cancer cell lines regardless of their RAS-RAF mutation status (Figure 1). Of greater interest, we found that this combination showed a synergistic antiproliferative activity and inhibition of angiogenesis in a colon cancer xenograft model with a bRAF mutation and multi drug resistant phenotype (Figure 4).

Initiation and progression of colorectal carcinoma is defined by abnormally high activation of RAS-RAF signaling pathway controlled by tyrosine kinases [18-20]. Activated forms of tyrosine kinases such as VEGFR, FGFR and PDGFR are known to play role in tumor angiogenesis, a process essential for growth of tumors. [22]. These receptors are activated by their corresponding growth factors secreted by tumor cells resulting in proliferation, migration and survival of tumor endothelial cells [49]. Although VEGF RTKs are the major targets for dovitinib, preclinical studies have also shown that FGF signaling is a possible mechanism of escape from and resistance to anti-VEGF therapy [50]. Therefore, dovitinib’s uniqueness in inhibiting growth factor receptors including FGFR and VEGFR makes it stand out among other RTKs inhibitors. A high percentage of colorectal carcinomas over-express a lot of growth factors and their receptors, including fibroblast growth factor (FGF) and FGF receptor (FGFR) [51]. Takayama *et al.* have shown that over-expression of FGFR correlates with liver metastasis in CRC [52]. Our results showed a decrease in phosphorylation of VEGFR and FGFR in two colon cancer cell lines tested. *In vitro* data in HCT116, HT-29 and SW-480 cell lines showed decreases in expression of all proteins in MAP kinase pathway such as kRAS, bRAF and pERK. Previous studies have shown that use of MEK inhibitors impaired proliferation thereby impacting a diverse array of cellular events, including differentiation, apoptosis, and angiogenesis [53]. However, Turke et al. have shown that MEK inhibitor led to activation of a parallel PI3K/AKT signaling pathway involving several feedback systems [54]. The *vice versa* has also been shown true in which inhibition of PI3K pathway activated MAP kinase pathway [55], thereby decreasing the effectiveness of single-agent targeted therapies. This suggests that concomitant inhibition of both pathways is necessary to block proliferation and induce cell death and shrink the tumor. Since inhibition by dovitinib in these cell lines was an upstream of kRAS, a parallel inhibition of both RAS-RAF-MAPK and PI3K-AKT suggest a synergistic effect of both pathways on downstream effectors of growth and proliferation. Our data also showed an inhibition of expression of pAKT in all three cell lines. Our results are in agreement with a recent report showing a concomitant down-regulation of PI3K and MEK induced regression of kRAS mutant cancers *in vivo*[56].

Our results with wound healing assay showed a significant decrease in wound repair with the use of combination of two as compared to either of the drugs alone confirmed a simultaneous inhibition of both signaling pathways (MAP Kinase and PI3Kinase) which are known to contribute to the inhibition of protein synthesis, cell growth, proliferation and survival. Lee et al. have shown that inhibition of FGFR and PDGFR starts as early as 4 h in the presence of Dovitinib [32].

A phase 1 pharmacokinetic and pharmacodynamic study of dovitinib in patients with advanced solid tumors showed a dose limiting toxicity of grade 3 hypertension and fatigue [57]. The strategy to improve the efficacy of the therapy and alleviate the symptom burden without increasing the toxicity is to add chemotherapeutic drug. In clinical studies, Oxaliplatin by itself has shown modest activity against advanced colorectal cancer. It has been extensively studied in combination with 5-FU and Folinic Acid. Our results demonstrate that when combined with dovitinib it showed a synergistic cytotoxicity by inducing apoptosis in colon cancer cell lines tested. There is compelling evidence that defects in apoptosis contributes to cancer. The molecular mechanism showed an increase in phosphorylation of histone H2AX at serine 139 in response to DNA double strand break by oxaliplatin. This DNA damage activated and stabilized p53, in turn, regulating the apoptotic pathway. It has been demonstrated many times that activation of p53 by DNA damage can lead to apoptosis by transcriptional activation of pro-apoptotic members of Bcl-2 family (Bax and Bak) and inhibition of anti-apoptotic (Puma, Noxa, Bcl2, Bclxl, Mcl-1 etc.) proteins, which together regulate mitochondrial permeability [58-60]. Also, it has been reported that AKT directly regulates members of the Bcl-2 super family and indirectly regulates apoptosis through the transcriptional factors that control apoptotic events [61]. Our results demonstrated an up-regulation of Bax and down-regulation of Bcl2 and Bclxl after treatment with the combination of oxaliplatin and dovitinib. The combination showed a more pronounced effect than either of the drugs alone. Mcl-1 a member of Bcl2 family and an inhibitor of apoptosis, showed a significantly higher expression in colon adenoma and carcinoma patient compared to healthy colonic epithelium [62]. Also, it has been shown that sustained activation of Akt resulted in increased expression of the antiapoptotic protein, Mcl-1 [63]. Our results showed a significant decrease in the expression of Mcl-1 after treatment with dovitinib possibly through the inactivation of AKT kinase. The expression was further reduced in the combination group in all three cell lines tested. Survivin is another molecule described to be involved in both the control of cell survival and regulation of cell cycle. Dramatic over-expression of Survivin compared with normal tissue has been shown in different kinds of cancer (reviewed in [64]). Survivin is also known to inhibit apoptosis mainly through targeting terminal effector caspase 3 activity in the apoptotic protease cascade [65]. Caspases are proteins known to be involved in the cascade of initiation and execution of apoptosis. Our results showed a decrease in Survivin after treatment with dovitinib and/or oxaliplatin in all cell lines. The combination treatment also showed a decrease in expression of procaspase 3, 8 and 9 with a subsequent increase in cleaved caspases. Our data also show a decrease and increase in expression of PARP and cleaved PARP respectively, a downstream target of activated caspase-3.

In parallel, *in vivo* results showed an inhibition of tumor growth in HT-29 tumor model with coordinating decrease in the expression of Ki-67 (biomarker for proliferation) and CD31 (biomarker for angiogenesis). The decrease was more pronounced in the combination group as compared to either of the groups alone. These results confirm that the combination inhibited angiogenesis which correlates to slow tumor growth supposedly because of lack of factors that are supplied through blood, thereby inhibiting the tumor growth. Our results are in agreement with previous reports showing inhibition of expression of Ki-67 and CD31 correlating to the shrinking of tumors and overall disease free survival [66,67].

## Conclusions

**Figure 5:**
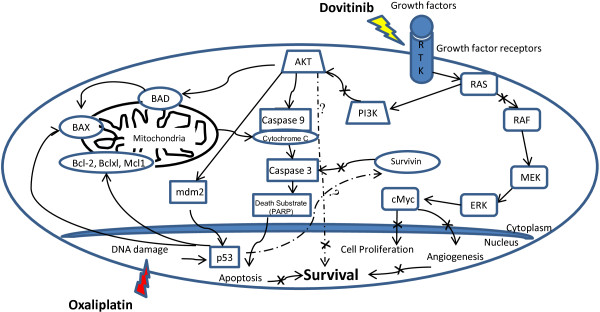
**A working model for the synergistic effect of dovitinib and/or oxaliplatin on tumor cells.** We propose that simultaneous inhibition of MAPK and PI3Kinase signaling pathways by dovitinib and DNA damage by oxaliplatin play an essential role in regulating apoptosis via induction of caspases cascade leading to a significant decrease in angiogenesis and inhibition of colon cancer cell proliferation.

Our results provide compelling evidence that dovitinib in combination with oxaliplatin inhibits cell growth and induces apoptosis in colon cancer cell lines. Simultaneous targeting of both MAP kinase and PI3Kinase by dovitinib to inhibit cell proliferation and induction of cell death through caspase dependent pathway by oxaliplatin contributes to the synergistic decrease in cell proliferation and viability. Furthermore, combined treatment with the two drugs effectively reduced growth of xenografted HT29 cells grown in athymic mice without exhibiting any toxicity in the animals. This antitumor efficacy of the combination was due to the inhibition of cell proliferation accompanied with suppression of angiogenesis. Schematic representation of our hypothesis is shown in Figure 5. In summary, combination of dovitinib and oxaliplatin produced a synergistic effect in colon cancer cells regardless of their RAS-RAF/p53 mutation status and also in a multidrug resistant clone of colon cancer model. These findings should be further explored in the clinic.

## Methods

### Materials

Human colon cancer cell lines HCT-116, HT-29, SW-480, Caco2 and LS-174 T were purchased from American Type Culture Collection (Manassas, VA). Cell culture media and serum were obtained from Invitrogen Life Technologies (Carlsbad, CA). Dovitinib (TKI-258) was obtained from Novartis (East Hanover, NJ) and Oxaliplatin was obtained from Sigma-Aldrich (St. Louis, MO). Antibodies against different proteins were obtained from Santacruz Biotechnologies Inc. (Santacruz, CA) or Cell signaling technology Inc. (Beverley, MA). HRP Conjugated anti-mouse IgG and Enhanced chemiluminescence plus (ECL plus) western blotting detection reagent were purchased from Amersham Bioscience (Arlington Heights, IL), X-OMAT AR films (Eastman Kodak, Rochester, NY). All other reagents were obtained from Fisher Scientific (Pittsburg, PA).

### Cell culture

The tumor cell lines were maintained in culture as adherent cells in a monolayer in humidified atmosphere at 37°C and 5% CO_2_ in McCoy’s 5A (HCT-116 and HT-29), Leibovitz’s L-15 Medium (SW-480), and Eagle’s Minimum Essential Medium (Caco2 and LS-174 T) and supplemented with 10% (20% for Caco2) heat-inactivated fetal calf serum. The cells were passaged twice a week and discarded after 20 passages.

### Cell viability assay

The cell viability was measured using MTS assay as described earlier [68]. IC_50_ values were calculated from the dose response curve generated from the colon cancer cell lines in the absence or presence of the drug(s). A real-time cell electronic sensing (RT-CES, ACEA Biosciences Inc., San Diego, CA) system was also used for measurement of cell response for combination of dovitinib and oxaliplatin in HCT-116, HT-29 and SW-480 cell lines. Briefly, 5000 cells were grown onto the surface of microelectronic sensors in a 16-well plate supplied by the manufacturer. After 24 h, a wide range of concentrations of drugs were added and the cells were continuously monitored by the system. The experiments were repeated with comparison of simultaneous and sequential addition of the two drugs. These results were further confirmed using MTS assay. Briefly, for combination experiments the drugs were mixed in 1:1 ratio of IC_50_ concentration or maximum achievable dosage and diluted to ½ and ¼ concentrations before the addition to the cells. Data from cell viability assay (MTS) were expressed as the fraction of cells killed by the individual drugs or the combination of drugs and compared to untreated cells.

### Determination of Synergism

The interaction between drug combinations was analyzed using Calcusyn software program (Biosoft, Cambridge, UK) to determine whether the combination was additive or synergistic. This program is based on Chou-Talalay method and calculates a combination index (CI), when CI =1, it indicates an additive effect and when below 1.0, it indicates synergism.

### Wound-healing assay

Cells were plated in 24-well plates and grown to confluence. The monolayer was wounded using the tip of a sterile 200 μl pipette. Cell debris was removed by washing twice with serum-free medium and replacing with medium containing serum and Dovitinib and/or Oxaliplatin. Cells were then allowed to migrate into the denuded areas for 24 hr. Photographs were taken immediately after wounding (t_0_) and 24 hr later (t_24_) using the Leica DMI3000 B inverted microscope. The results were quantified as a percentage of the wound width closed by the cells at time 24 hr (T_24_ = (100/t_0_)/t_24_). The mean of three experiments was graphed with standard deviations represented as error bars.

### Western blot analysis

Cells were collected after 24 h treatment with dovitinib and oxaliplatin and washed once with PBS and second time with cold PBS containing 0.1 mM orthovanadate. The whole cell lysates were prepared according to the procedures described previously [68]. Protein was measured using Bio-Rad protein assay kit (Bio-Rad, Hercules, CA) and. The proteins were transferred to PVDF membrane (Amersham, Arlington Heights, IL) after resolving by (25 μg protein per lane) 4-12% gel electrophoresis (SDS-PAGE) and probed with one of the following: RAS, RAF, p-ERK, ERK, p-AKT, AKT, Survivin, Caspase 3, Caspase 9 (Cell signaling technology, Boston, MA), p53, Anti-phosphotyrosine (4G10), γH2AX and pATM, β-actin (EMD Millipore, Billerica, MA ), p21, Bax, Bcl-2, Bcl_xl_, Mcl-1, cytochrome C, c-Myc (Santa Cruz biotechnology, Santacruz, CA), GAPDH (GeneTex, Irvine, CA) and Cleaved PARP (Promega, Madison, WI) antibodies and HRP conjugated secondary antibody (Amersham). The optical density for each band was determined using Image Quant software (GE Healthcare Biosciences, Pittsburg, PA).

### Apoptotic cell death assay

To quantify apoptosis, HCT-116, HT-29 and SW-480 cells were stained with annexin V and PI using FITC Annexin V Apoptosis detection kit 1 from BD Pharmingen following the step by step protocol as provided by the manufacturer and analyzed by flow cytometry (BD Bioscience, San Diego, CA). Briefly, at the end of treatment with dovitinib and oxaliplatin either alone or in combination for 48 h, both floating and attached cells were collected, washed twice with cold PBS and subjected to annexin V-FITC and PI staining and analyzed using flow cytometry.

### Subcutaneous human tumor xenograft

*In vivo* evaluation of Dovitinib and/or Oxaliplatin in HT-29 human colorectal cancer model was performed at Institute of Translational Medicine, Taipai Medical University (TMU), Taipei, Taiwan.

### Animal

Forty female athymic nude mice (BALB/cAnN.Cg-*Foxn1*^*nu*^/CrlNarl; 5 weeks of age) were purchased from the NAR Labs National Laboratory Animal Center (Taipei, Taiwan). Mice were housed in TMU Laboratory Animal Center (Taipei, Taiwan) around a specific pathogen-free animal facility at constant temperature (20 ± 3°C) and humidity (50 ± 20%). The animals had free access to irradiation-sterilized dry granule food and water during the study period. Animal care and the treatment were performed according to the guidelines of the Institutional Animal Care and Use Committee (IACUC) based on guidance of the Association for Assessment and Accreditation of Laboratory Animal Care (AAALAC).

### Cell culture

HT-29 tumor cells were maintained *in vitro* in McCoy’s 5A medium supplemented with 10% FBS and 0.1 mM NEAA. The cells growing in exponential growth phase were harvested and counted for tumor inoculation.

### Tumor inoculation

Each mouse was inoculated subcutaneously at the rear right flank with HT29 tumor cells (3 × 10^5^) in 0.1 ml of PBS for tumor development. After 10 days of tumor inoculation, the animals were weighed and measured for tumor volume and randomly divided into 4 groups of 10 animals each based on the randomized block design method for homogeneous group formation when the mean tumor size reached approximately 80–125 mm^3^ (10 Days).

### Drug treatment

The treatment was started intravenously (*i.v.*) on the 11^th^ day post tumor inoculation with 0.9% saline (Group 1), 6.7 mg/Kg Oxaliplatin (Group 2), 60 mg/Kg Dovitinib (Group 3) and 6.7 and 60 mg/Kg Oxaliplatin and Dovitinib (Group 4) respectively. The treatment was continued for 3 weeks with a regimen of once per week (i.v.) for Oxaliplatin and every two days (oral) for Dovitinib.

### Tumor measurement

The animals were visually monitored for food and water consumption everyday and once/week for body weight (gain/loss) and tumor size. Tumor volumes were calculated using formula: V = 0.5 × a × b^2^ where a and b are the long and short tumor diameters respectively and euthanized when the tumor volume reached a predetermined size of approximately 3000 mm^3^. This end-point tumor size was chosen to maximize the number of tumor doublings within the exponential growth phase in the untreated group. All the tumors were harvested a week after the last treatment and fast frozen for immunohistochemistry.

### Tissue preparation and immunohistochemical staining

The immunohisto-chemical assays were performed using a Dako Autostainer Plus (Dako Colorado Inc., CO) with fresh sections of vehicle control and treated tissue stained at the same time with the help of research pathology core facility at the City of Hope as described in [69]. Primary rabbit Ki67 (Novus Biologicals) or mouse monoclonal CD31 antibody (Cell Marque, CA) were used for IHC at a final concentration of 1:100(Ki-67) or 1:75 (CD31). The sections were counterstained with Meyer’s haematoxylin and each run also included phosphate buffered solution (PBS) used as the primary antibody for the negative controls while samples known to express Ki-67 or CD31 strongly served as positive controls. Photomicrographs were taken on a Nikon microscope equipped with a CCD camera.

### Statistical analysis

Data points for cell proliferation and apoptosis were presented as mean ± standard error mean of at least three independent cell populations. Results were compared using two-tailed student’s t-test using Microsoft Excel Program 2003. A p-value <0.05 was considered statistically significant. Animal data results were compared using student’s t-test. Animal study data were evaluated using one-way ANOVA followed by Dunnett’s post-test if significance was observed. The data were analyzed using SPSS version 16.0. A p-value <0.05 was considered statistically significant.

## Abbreviations

CRC: Colorectal carcinoma; VEGF: Vascular endothelial growth factor; VEGFR: Vascular endothelial growth factor receptor; FGF: Fibroblast growth factor; FGFR: Fibroblast growth factor receptor; PDGF: Plate-derived growth factor; RTK: Receptor tyrosine kinase; RAS: Rat sarcoma; RAF: Rapidly activated fibrosarcoma; MAPKinase: Mitogen activated protein kinase; ERK: Extracellular signal-regulated kinase; AKT (PKB): Protein kinase B; PIP2: Phosphatidylinositol; PIP3: (4,5)-bisphosphate phosphatidylinositol (3,4,5)-trisphosphate; PH: Pleckstrin homology; 5FU: 5-fluorouracil; WNT: Mammalian homolog of Wingless (Wg); APC: Adenomatous polyposis coli; TKI: Tyrosine kinase inhibitor; c-KIT: Tyrosine-protein kinase kit; FLT-3: Fms-like tyrosine kinase 3; PI3K: Phosphoinositide 3 kinase; p53: Tumor protein 53; p21: Protein 21; IC50: Half maximal inhibitory concentration; RT-CES: Real time cell electronic sensor; MTS: 3-(4,5-dimethylthiazol-2-yl)-5-(3-carboxymethoxyphenyl)-2-(4-sulfophenyl)-2H-tetra-zolium; CI: Combination index; MEK: Mitogen activated protein kinase kinase; DSB: Double strand break; DNA: Deoxyribonucleic acid; H2AX: H2A histone family, member X; Bcl2: B-cell lymphoma 2 protein; Bax: Bcl2 associated X protein; Bclxl: B-cell lymphoma extra large; Mcl-1: Induced myeloid leukemia cell differentiation protein; PI: Propidium Iodide; AD Ribose: Adenosine ribose; PARP: Poly ADP ribose polymerase; DT: Doubling time; CD-31: Cluster of differentiation -31; Ki-67: Antigen Ki-67; mm: Millimeter; Kg: Kilogram; ECL: Enhanced chemiluminescence; Nm: Nanometer; t0: Time at 0 hour; t24: Time at 24 hour; μg: Microgram; μM: Micromolar; PBS: Phosphate buffer saline; HRP: Horse radish peroxidase; FITC: Fluorescein isothiocyanate; FBS: Fetal bovine serum; i.v.: Intravenous; ANOVA: Analysis of variance; CCD camera: Charge-coupled device camera.

## Competing interests

The authors declare that they have no competing interests.

## Authors’ contributions

SG and YY designed the study. SG, LC, VA, WL and YW performed the experiments. VC, NYH and HSS executed the animal experiments. SG and YY analyzed the data and drafted the manuscript. All authors read and approved the final manuscript.
